# Study on Configuration Design and Numerical Simulation of Twin-Screw Extruder Cooling Die Based on Pea Protein Isolate Flow Properties

**DOI:** 10.3390/foods14173137

**Published:** 2025-09-08

**Authors:** Miao Yang, Xun Zhang, Min Wu, Tianqi Zhang, Fanglei Zou, Shuqi Yang

**Affiliations:** College of Engineering, China Agricultural University, Beijing 100083, China; myang@cau.edu.cn (M.Y.); zhangxun@cau.edu.cn (X.Z.); sy20233071641@cau.edu.cn (T.Z.); flzou@cau.edu.cn (F.Z.); yang.shv@foxmail.com (S.Y.)

**Keywords:** pea protein isolate, twin-screw extruder, cooling die, configuration design, numerical simulation

## Abstract

This study developed an optimized cooling die configuration to improve the fibrous structure and texture of protein extrudates. Six designs, combining three cross-sectional shapes and two flow channel layouts, were evaluated through numerical simulations based on the physical properties of pea protein isolate (PPI) and extrusion parameters. The results show that PPI exhibits pronounced shear-thinning behavior, with viscosity decreasing by more than 85% as the temperature increases from 35 °C to 135 °C. Among all designs, the rectangular outlet with a serpentine cooling channel performed best, showing a center-to-wall temperature difference of 12.4 °C compared with 7.8 °C for the circular die, a 35% higher heat transfer coefficient, a wall-to-center viscosity ratio of 7.4 compared with 4.9 for the square die and 3.7 for the circular die, and a maximum wall shear rate of 3.42 s^−1^ compared with 2.15 s^−1^ for the circular die. The rectangular outlet increases the center-to-wall temperature gradient, while the serpentine channel extends the flow path to raise shear and velocity gradients, together promoting fiber alignment and improving the structure of plant-based meat. These findings provide a theoretical foundation for cooling die optimization and offer a practical approach to control fiber formation in plant-based meat.

## 1. Introduction

With the global population steadily increasing, the imbalance between meat supply and demand has become more pronounced. Expanding non-animal protein sources is now a critical challenge in the food industry [1]. Plant-based meat, primarily made from proteins such as soy protein isolate (SPI) and pea protein isolate (PPI), aims to replicate the sensory and nutritional qualities of animal meat [2]. Compared to SPI, PPI offers greater commercial potential due to its superior nutritional value, low allergenicity, and functional versatility [3].

High-moisture extrusion (HME) has emerged as a core technology for producing plant-based meat due to its maturity, efficiency, and wide applicability. Under high temperature and moisture, this process enables protein molecular rearrangement, followed by cooling and shaping to form fibrous textures [4,5,6]. Compared to a single-screw extruder, the twin-screw extruder enables more precise control over mixing, heating, and shear [7,8]. Based on the structure and function of different sections, the extruder is typically divided into the feeding zone, mixing zone, melting zone, die zone, and cooling zone [9]. The cooling die, a critical part of the cooling zone, determines the temperature drop and structural alignment of proteins, directly impacting final texture [10,11]. A higher temperature gradient in the cooling die results in less pronounced protein gelation, leading to a softer texture while promoting the alignment of molten protein material, significantly enhancing the texturization index of protein extrudates [3]. Although previous studies have made initial explorations into the design of extruder cooling die configurations [12,13,14], the quantitative coupling between PPI rheology and die geometry/layout remains limited. Prior works rarely compare iso-area cross-sections with distinct cooling-channel layouts under non-isothermal conditions.

Typical cooling die configuration parameters include channel cross-sectional shape, distribution of cooling media, number and arrangement of flow paths. These factors directly influence the velocity gradient, cooling rate, and fiber formation tendency of the material within the die [12,14]. PPI exhibits non-Newtonian shear-thinning behavior during HME, with its rheological properties highly sensitive to temperature, shear rate, and moisture content. In the cooling die, rapid solidification from molten to gel-like states, governed by complex thermo-fluid-structure coupling, makes protein flow and molding highly dependent on die configuration [15,16]. However, quantitative research on how these factors influence fiber formation remains limited.

Numerical simulation tools provide dynamic, real-time modeling of flow and thermal behaviors [17,18,19]. Sun et al. [18] utilized these tools to investigate the influence of screw parameters on protein structure formation, while Cheng et al. [20] developed a simulation model based on melt rheological properties to predict expansion characteristics under various formulations in the twin-screw extrusion process. Numerical simulations are useful not only for optimizing experimental parameters and revealing property change mechanisms but also for optimizing extruder structure and die design. For example, they are applied in screw parameter optimization [21], screw element combination optimization [22], and die structure optimization [23]. Nevertheless, numerical simulations have not yet been applied to cooling die design.

This study addresses this gap by combining theoretical analysis with numerical simulations to design and optimize cooling die configurations for PPI. It establishes design criteria based on material and process properties and evaluates six die structures under non-isothermal conditions. The results reveal the molding mechanisms during cooling and provide a foundation for controlled fiber formation in plant-based meat, thereby promoting sustainable and healthy food choices while supporting the industrialization of plant protein-based products.

## 2. Materials and Methods

### 2.1. Materials

The PPI used in the experiments was purchased from Yantai Shuangta Food Co., Ltd. (Yantai, China) with a protein content of 82.01 ± 0.54%.

### 2.2. Density Measurement of PPI

The density of the pasty PPI with a moisture content of 60% was measured using the gravimetric bottle displacement method at temperatures of 35 °C, 45 °C, 55 °C, 65 °C, 75 °C, and 85 °C. Specifically, 1 g of the pasty PPI sample, preheated to the corresponding temperature, was placed in a pycnometer. The sample was then equilibrated in a water bath for 3 min before measuring its density. Triplicate measurements were performed at each temperature to ensure the accuracy and consistency of the results.

### 2.3. Viscosity Measurement of PPI

The viscosity of pasty PPI samples with 60% moisture content was measured at various temperatures using a closed cavity rheometer (MCR302, Anton Paar GmbH, Graz, Austria). Temperature was varied from 35 °C to 145 °C at 20 °C intervals, while shear rate was gradually increased from 0.1 to 100 s^−1^ under a constant pressure of 0.4 MPa, which matched the pressure monitored during the actual extrusion process. Measurements recorded by the instrument software yielded parameters including shear force, shear rate, and apparent viscosity of the PPI paste under different conditions. These data were collected and fitted to the Power-Law rheological model using Origin Pro 2025 software to establish the rheological model for pasty PPI. The fitting formula is as follows. The fitting formula is given in Equation (1) [24].

(1) $$\eta =K\cdot \gamma^{n-1}$$

where *η* is the apparent viscosity, *K* is the viscosity index with the unit of Pa⋅s*^n^*, and *n* is the flow index.

### 2.4. Determination of Thermodynamic Parameters of PPI

The moisture content of the PPI sample was adjusted to 60%. During testing, the sample was allowed to stabilize at the set temperature for 30 min, with temperature fluctuations maintained below 1 °C. Following this stabilization period, a thermal constant analyzer (TPS3500, Hot Disk AB, Gothenburg, Sweden) was used to measure and record the specific heat capacity and thermal conductivity (λc) of the sample under different temperature conditions, at a pressure of 0.4 MPa, consistent with the die inlet pressure in the extrusion process.

### 2.5. Design Requirements for Twin-Screw Extruder Cooling Die

The design of cooling dies must comprehensively account for material properties, structural layout, and heat exchange efficiency. For material selection, priority should be accorded to materials exhibiting high strength, high thermal conductivity, and corrosion resistance. Dimensional design must ensure robust compatibility with the barrel while integrating considerations of structural strength, heat dissipation performance, flow stability, and cooling efficiency; these factors are all pivotal to sustaining stability and molding quality in PPI extrusion processes. Structurally, a compact layout is essential: cooling units should be closely arranged to shorten heat transfer paths and maximize contact area with cooling water, thereby enhancing heat exchange efficiency. Cooling water channels require smooth, streamlined configurations to avoid abrupt transitions and dead zones, ensuring stable coolant flow and uniform coverage of the melt. In terms of geometry, symmetrical structures are preferred as they facilitate uniform heat distribution, allowing the melt to reach an optimal state prior to molding. This, in turn, promotes fiber formation and improves product quality consistency.

### 2.6. Material Flow Equation for Twin-Screw Extruder

The material flow rate in a twin-screw extruder, which is derived from screw geometry parameters, can be expressed by Equation (2) [25].

(2) $$\;Q=\alpha n-\beta \frac{p}{\eta_{1}}-\gamma \frac{p}{\eta_{2}}=\alpha n-(\frac{\beta }{\eta_{1}}+\frac{\gamma }{\eta_{2}})P$$

where *α*, *β*, and *γ* are constants, their specific expressions are respectively listed in Equations (3)–(5).

(3) $$\alpha =\frac{1}{2}\pi^{2}D^{2}H\cos\varphi \sin\varphi$$

(4) $$\beta =\frac{\pi DH^{3}\sin^{2}\varphi }{12L^{3}}$$

(5) $$\gamma =\frac{\pi^{2}D^{2}\delta^{3}\tan\varphi }{10bL^{3}}$$

where *D* is the screw outer diameter, *L* is the effective length of the screw, *H* is the groove depth of the metering section, *φ* is the screw thread helix angle, *δ* is the gap between the barrel and the screw, *p* is the die pressure, *η*_1_ is the viscosity of the molten material in the screw groove, and *η*_2_ is the viscosity of the molten material in the gap *δ*. The values of *η*_1_ and *η*_2_ are determined based on the rheological properties of the material.

Once the extrusion process reaches a steady state, both temperature and screw speed can be considered as constant parameters. Therefore, under these conditions, the viscosities *η*_1_ and *η*_2_ can also be approximated as constants. Based on these assumptions, a linear relationship exists between the volumetric flow rate *Q* and the pressure *P*. The relationship between the flow rate through the die and the pressure drop across it is given by Equation (6) [26].

(6) $$Q=K_{Q}\frac{\Delta P}{\eta_{3}}$$

where *K_Q_* is the die shape factor, Δ*P* is the pressure drop across the die, and *η*_3_ is the viscosity of the material within the die, which can be determined based on the rheological properties of the material.

### 2.7. Die Flow Channel Design for Twin-Screw Extruder

**Figure 1:**
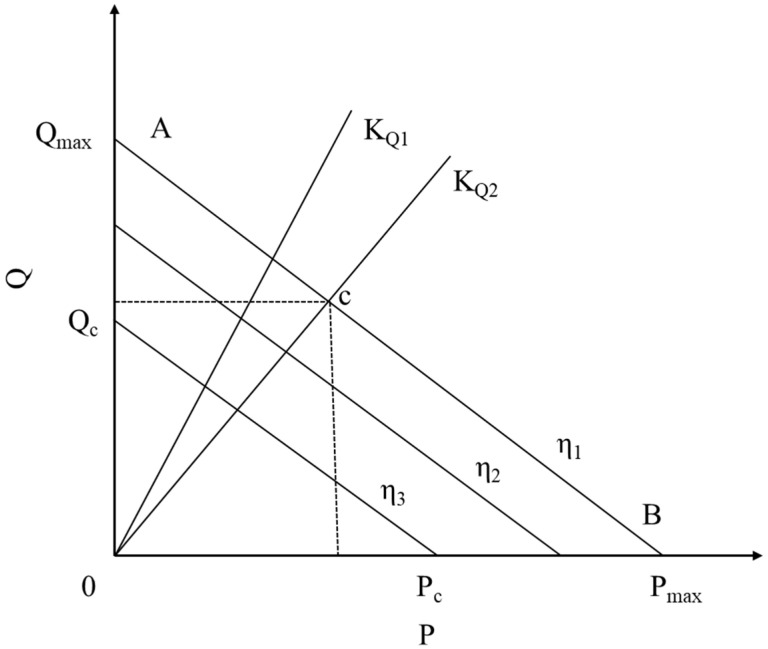
Twin screw extruder screw and die port characteristic lines.

As shown in Figure 1, the die characteristic line *K_Q_* and screw characteristic line AB are plotted using Equations (7) and (8), respectively. Their intersection c denotes the extruder’s operating point, corresponding to die pressure *P_c_* and volumetric flow rate *Q_c_.* With the die outlet pressure being 0, Δ*P* can be assumed equal to *P_c_*. Solving these two equations simultaneously gives the solution derived from Equations (7) and (8) [26].

(7) $$P_{c}=\frac{\alpha n\eta }{K_{Q}+\beta +\gamma }$$

(8) $$Q_{c}=\frac{K_{Q}\alpha n}{K_{Q}+\beta +\gamma }$$

where simplification is applied, the material viscosity of the screw and die is assumed equal, denoted as *η*.

The die characteristic factor *K_Q_* can be determined based on the die shape. The characteristic factors for common die shapes given in Equations (9)–(12).

Narrow flat-slot die:

(9) $$K_{Q}=\frac{bH^{3}}{12L}$$

Cylindrical die:

(10) $$K_{Q}=\frac{\pi R^{4}}{8L}$$

Conical die:

(11) $$K_{Q}=\frac{3\pi D^{3}d^{3}}{128(D^{2}+Dd+d^{2})}$$

Annular die:

(12) $$K_{Q}=\frac{\pi }{8L}\left[R^{4}-r^{4}-\frac{\left(R^{2}-r^{2}\right)}{2.31og10(\frac{R}{r})}\right]$$

### 2.8. Three-Dimensional Modeling and Meshing of Cooling Die

**Figure 2:**
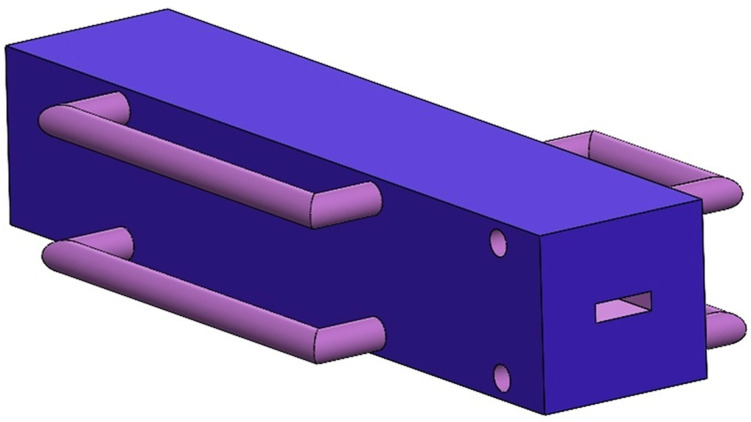
Schematic of 3D modeling of cooling die.

The die design schemes were modeled in 3D, with the cooling die created using the professional modeling software SolidWorks 2022. The die’s external dimensions are 318 mm × 70 mm × 65 mm. The cooling water flow channels are arranged in serpentine and straight-line configurations. The internal exit cross-sectional dimensions of the three protein melt flow channels are as follows: rectangular (24 mm × 7 mm), circular (φ, diameter = 14.64 mm), and square (12.96 mm × 12.96 mm). The modeling results are shown in Figure 2.

**Figure 3:**
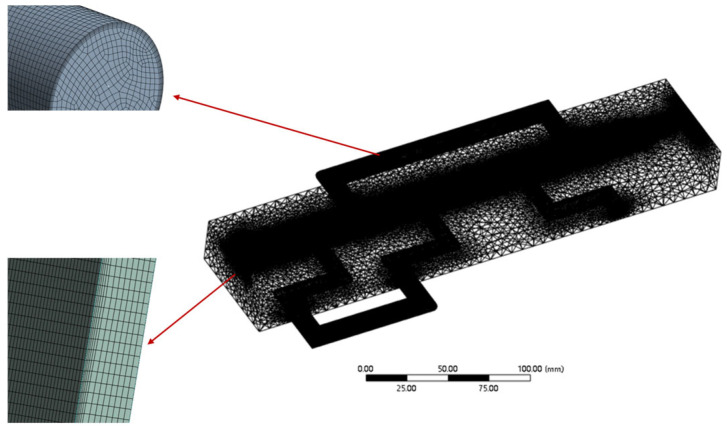
Schematic mesh delineation of cooling die.

Meshing was performed using Ansys Mesh 2024 R2. Due to the symmetric design of the upper and lower parts of the 3D model, symmetry was employed in the simulation to simplify calculations. After a mesh independence test, the number of meshes in the computational domain was approximately 3.9 million. The minimum orthogonal quality of the meshes was 0.39, with over 90% exceeding 0.5, indicating that the mesh quality met the requirements. Furthermore, to ensure accurate calculation of the flow process, hexahedral meshes were used for the molten protein fluid region and cooling water channels, with boundary layers added (5 layers in total). The meshing of the cooling die is shown in Figure 3.

Mesh independence was verified by comparing results from coarse (0.42 million elements), medium (0.78 million elements), and fine (1.15 million elements) meshes. The outlet average velocity and centerline temperature differed by less than 2.5% between the medium and fine meshes, while the computational time increased by over 45%. Therefore, the medium mesh was considered sufficiently accurate and was used in all subsequent simulations (Table 1).

### 2.9. Basic Assumptions of Flow Field

The actual extrusion and cooling process of PPI melt is quite complex. On one hand, this complexity arises from the intricate structure of the die flow channels. On the other hand, it is influenced by the variable physicochemical properties of PPI under multiple physical field conditions. To facilitate mathematical description and simplify iterative computations and solutions, the following assumptions are made based on the characteristics of the protein fluid and the cooling die:

(1) During the cooling process, the PPI melt is assumed to fill the entire cooling die uniformly;
(2) No slip occurs at the cooling die wall, i.e., the no-slip condition is applied at the wall;
(3) The protein fluid is considered to flow in a laminar manner;
(4) The effects of inertial forces, gravity, and other volumetric forces are neglected, as viscous forces are much greater than these;
(5) No phase change occurs in the protein fluid during the cooling process.

### 2.10. Fluid Control Equations

The flow of PPI within the flow channels is described using fluid dynamics equations, specifically subdivided as follows [27].

(1) Continuity Equation

The flow expression for the fluid in a Cartesian coordinate system is given by Equation (13).

(13) $$\frac{\partial \left(\rho u_{x}\right)}{\partial x}+\frac{\partial \left(\rho u_{y}\right)}{\partial y}+\frac{\partial \left(\rho u_{z}\right)}{\partial z}+\frac{\partial \rho }{\partial t}=0$$

where the fluid is assumed incompressible with a constant *ρ*, Equation (13) simplifies to Equation (14).

(14) $$\frac{\partial u_{x}}{\partial x}+\frac{\partial u_{y}}{\partial y}+\frac{\partial u_{z}}{\partial z}=0$$

By rearranging Equations (13) and (14), the following can be obtained.

(15) $$\frac{\partial \rho }{\partial t}+\nabla \cdot \left(\rho u\right)=0$$

where *ρ* represents density with units of kg/m^3^, and *u* represents velocity with units of m/s.

(2) Momentum Equation

The momentum equation, derived from Newton’s second law, results in the final expression given by Equation (16).

(16) $$\rho \frac{\partial u}{\partial t}+\rho \left(u\cdot \nabla \right)u=\nabla \cdot \left(-p+S-\frac{2}{3}\eta \left(\nabla \cdot u\right)I\right)+F$$

where *ρ* is the density with units of kg/m^3^; *u* is the velocity with units of m/s; *p* is the pressure with units of Pa; *η* is the dynamic viscosity with units of Pa·s; *F* is the volumetric force with units of N/m^3^; and *S* is the additional stress tensor with units of N/m^2^.

The formula is given in Equation (17).

(17) $$S=\eta (\nabla u+{\left(\nabla u\right)}^{T})$$

where *T* represents the absolute temperature with units of K.

(3) Energy Equation

The formula is given in Equation (18).

(18) $$\rho c_{p}\frac{\partial T}{\partial t}+\rho c_{p}u\cdot \nabla T+\nabla \cdot q=Q$$

where *c_p_* is the specific heat capacity with units of J/(kg·K); *Q* is the internal heat source with units of W/m^3^; *q* is the heat flux with units of W/m^2^. From Equation (18), the following can be obtained:

(19) q=−k∇T

where *k* is the heat transfer coefficient with units of W/(m^2^·K).

The extrusion mass flow rate *Q* is obtained by integrating the axial velocity *v_z_* inside the cross-section of the cooling die, and the specific formula is given in Equation (20).

(20) $$Q=\iint v_{z}ds$$

Shear stress is given by Equation (21).

(21) $$\sigma_{s}=\eta \sqrt{{\left(\frac{\partial \bar{v}}{\partial x}+\frac{\partial \bar{w}}{\partial x}\right)}^{2}+{\left(\frac{\partial \bar{u}}{\partial y}+\frac{\partial \bar{w}}{\partial y}\right)}^{2}+{\left(\frac{\partial \bar{u}}{\partial z}+\frac{\partial \bar{v}}{\partial z}\right)}^{2}}$$

### 2.11. Setting of Boundary Conditions

This study focuses on the twin screw extruder (TwinLab-F 20/40, Brabender, Oberhausen, Germany). In this extruder, the hopper and water inlet are located 3 cm and 24 cm from the start of the screw, respectively. The screw has a length-to-diameter ratio of 40:1 and a diameter of 20 mm.

The boundary conditions are determined based on the specific conditions of the extruder and the experiment: The wall temperature is set to follow the heat conduction direction perpendicular to the wall. The initial temperature of the molten protein entering the die is set to 130 °C, and the cooling water temperature entering the cooling channels is set to 65 °C. The cooling water inlets on the top and bottom sides of the cooling die are set as velocity inlets, with an inlet velocity of 0.5 m/s. The initial pressure of the molten protein fluid entering the die is set to 0.4 MPa. Both the cooling water outlet and the molten protein fluid outlet are set as pressure outlets with no backflow, and the pressure value is set to 0.

### 2.12. Statistical Analysis

All experiments were run in triplicate, with results expressed as mean ± standard deviation unless specified otherwise. Statistical analysis was performed using SPSS 20.0 (SPSS Inc., Chicago, IL, USA) with one-way analysis of variance (ANOVA), and significant differences between samples (*p* < 0.05) were identified via Duncan’s multiple range test.

## 3. Results and Discussion

### 3.1. Determination of PPI Density

**Figure 4:**
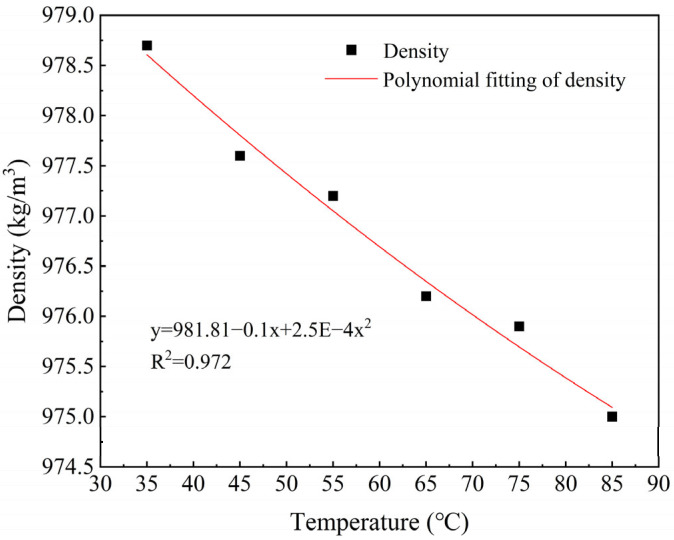
Changes in density of PPI paste at different temperatures.

Figure 4 shows the density variation of PPI paste with a moisture content of 60% in the temperature range from 35 °C to 85 °C, along with the corresponding quadratic polynomial fitting curve. The results indicate that as the temperature increases, the density of the system exhibits a nonlinear decreasing trend. Specifically, the density decreased from 978.72 kg/m^3^ at 35 °C to 975.16 kg/m^3^ at 85 °C, showing an overall reduction of about 0.36%. Under the conditions of higher moisture content (60%), the protein undergoes structural expansion and partial denaturation due to heat. This process forms a network with a looser structure. As a result, the molecular density decreases, and consequently, the overall density is reduced [28].

### 3.2. Determination of PPI Viscosity

**Figure 5:**
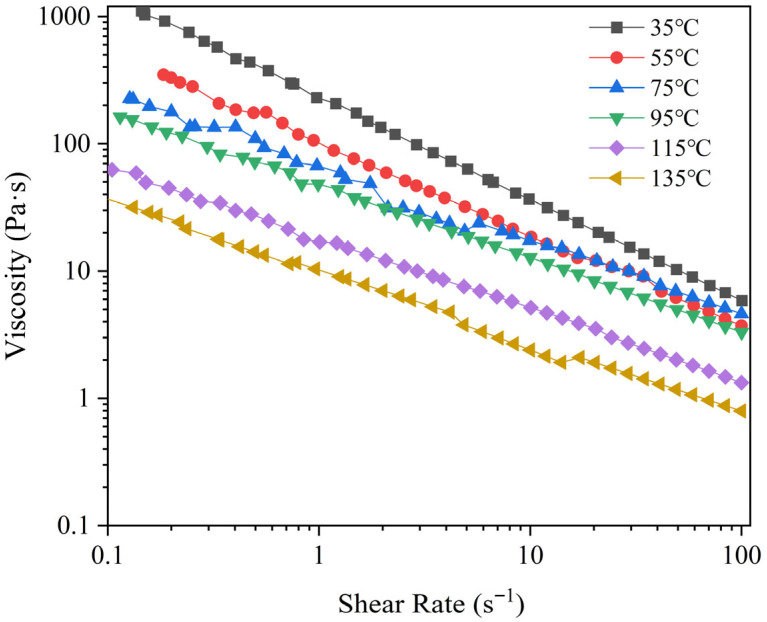
Variation of apparent viscosity of PPI paste in relation to shear rate at different temperatures.

The relationship between the viscosity and shear rate of PPI paste with a moisture content of 60% at different temperatures (35 °C to 135 °C) is shown in Figure 5. The results indicate that under all temperature conditions, the apparent viscosity of the sample decreases significantly as the shear rate increases. This phenomenon exhibits typical shear-thinning behavior. It also shows characteristics of a pseudoplastic fluid [29,30]. In the low shear rate range (0.1–10 s^−1^), the viscosity decreases rapidly, while as the shear rate further increases (10–100 s^−1^), the viscosity change becomes more gradual. This behavior may be related to the dynamic equilibrium between the orientation and structural breakdown of the PPI molecular chains. On the other hand, temperature variation has a significant impact on the viscosity of PPI. At the same shear rate, the viscosity decreases as the temperature increases. For example, at 35 °C, the apparent viscosity of PPI ranges from 1104.17 Pa·s to 5.85 Pa·s as the shear rate increases from 0.1 s^−1^ to 100.0 s^−1^. At 135 °C, the apparent viscosity range drops to 38 Pa·s to 0.79 Pa·s. This suggests that high temperatures accelerate the weakening of intermolecular interactions in PPI, reducing the system’s flow resistance. This phenomenon is consistent with the findings of Jebalia et al. [31].

The Power-Law model *K* and *n* values for PPI at different temperatures are shown in Table 2. The results indicate that all n values are less than 1 (ranging from 0.20 to 0.43), further confirming the shear-thinning characteristics of the sample [32]. As the temperature increases (from 35 °C to 135 °C), the *K* value significantly decreases, from 236.10 Pa·s to 9.84 Pa·s (*p* < 0.05), indicating that high temperatures cause the PPI network structure to become more loose, resulting in a reduction in viscosity [33].

### 3.3. Determination of Thermodynamic Properties of PPI

**Figure 6:**
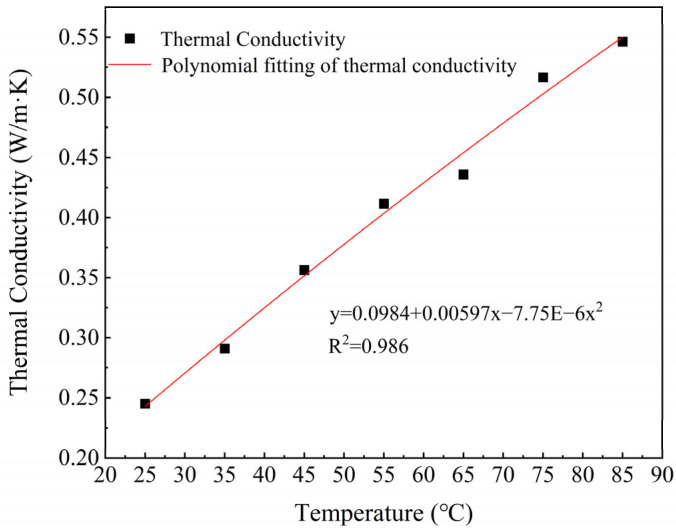
Variation of thermal properties of PPI with thermal conductivity.

Figure 6 shows that the thermal conductivity of PPI increases nonlinearly with increasing temperature. This trend is consistent with the research by Elisabeth et al. [34] on the thermal conduction behavior of SPI. This nonlinear response can be attributed to the enhancement of hydrophobic cross-linking and molecular rearrangement during protein thermal denaturation. This enhancement, in turn, induces the formation of aggregates. It also widens the heat conduction pathways [35,36].

**Figure 7:**
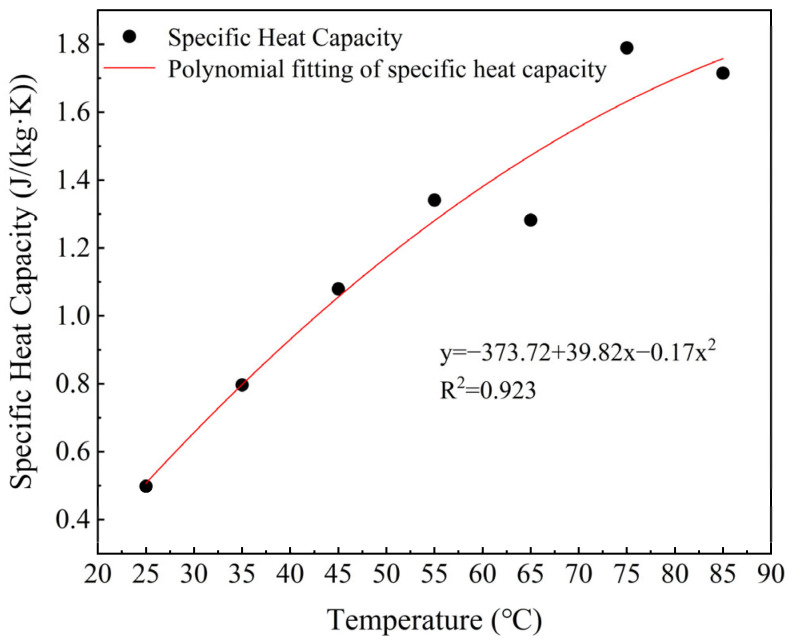
Variation of thermal properties of PPI with specific heat capacity.

Figure 7 shows that the specific heat capacity increases continuously within the measured temperature range (20–120 °C). This indicates that the increase in temperature promotes the thermal motion of molecular segments. As a result, the system needs to absorb more energy to achieve the same temperature increment [37]. The protein-water interactions under high moisture conditions also enhance the thermal buffering capacity of the system, leading to a further increase in specific heat capacity [34].

### 3.4. Die Design Scheme

#### 3.4.1. Material Selection for the Die

The high-strength properties of the cooling die material are crucial under long-term use and high-intensity operating conditions. Additionally, the material must have a high yield strength and excellent corrosion resistance to withstand humid and hot conditions while reducing production costs. Although 304 stainless steel is cost-effective, its thermal conductivity is insufficient to meet the demands for efficient heat dissipation [38]. The die material must possess high thermal conductivity to fully leverage the cooling medium and promote fiber formation in the product [38,39]. 17-4PH stainless steel retains the advantages of traditional stainless steel while additionally boasting excellent thermal conductivity and corrosion resistance, thus rendering it an ideal material for manufacturing cooling dies [40,41].

#### 3.4.2. Determination of Die Geometric Dimensions

**Figure 8:**
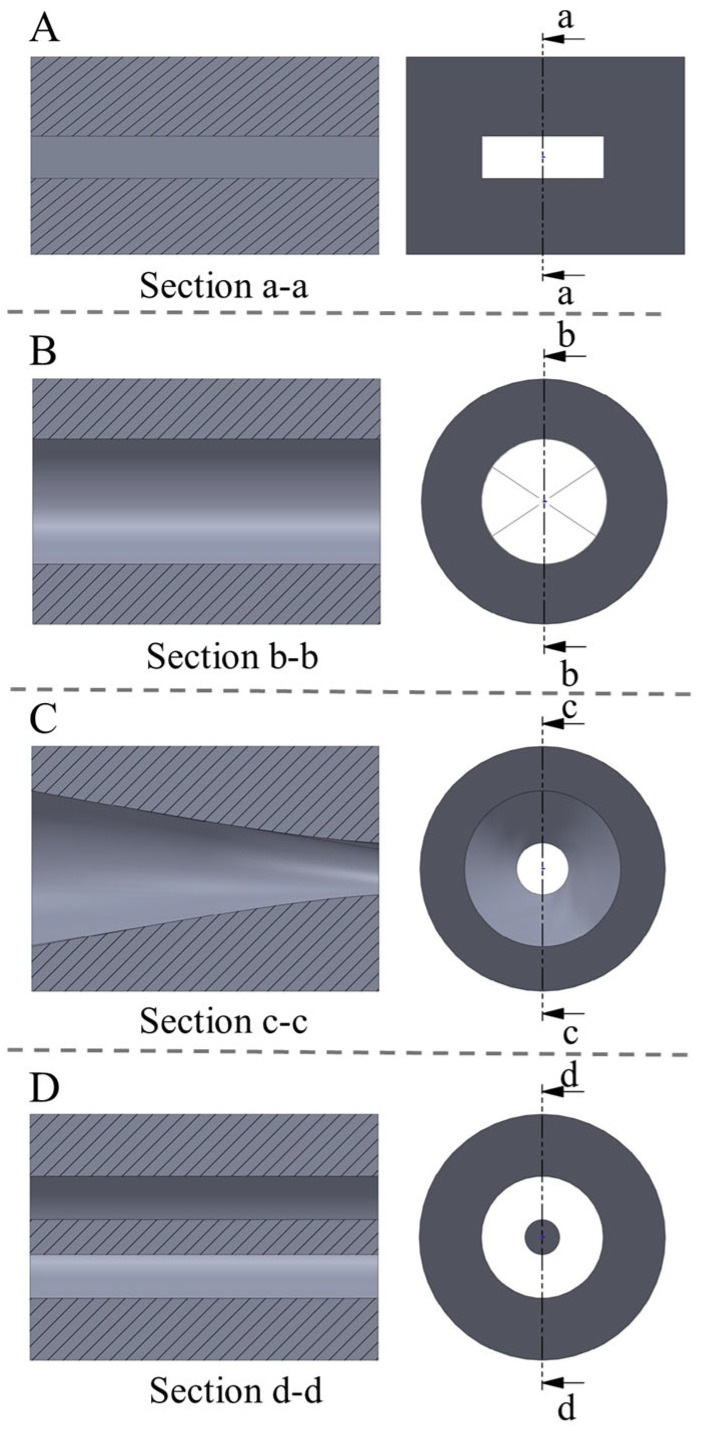
Several common mouth mold shapes. (**A**) narrow flat seam type, (**B**) cylindrical type, (**C**) conical type, and (**D**) circular type.

To ensure the geometric compatibility of the die with the twin-screw extruder system, the connection dimensions should be based on the barrel inner diameter design to ensure a proper fit. The outer diameter of the die body must be determined in conjunction with the die cross-sectional shape, such as rectangular, circular, or custom shapes. It needs to balance structural strength and heat dissipation performance. This avoids thermal deformation caused by a too-small diameter or material waste due to an excessively large diameter. The length of the die body should consider both cooling rate and pressure drop control. A length that is too short is detrimental to fiber structure formation. Conversely, a length that is too long increases flow resistance [42]. Four die mouth shapes were designed, as shown in Figure 8.

#### 3.4.3. Layout Scheme of Die Cooling Channels

The arrangement of cooling channels is a key aspect of cooling die design. The core objective is to achieve efficient heat exchange between the high-temperature material and the cooling medium by optimizing the spatial layout of the channels and the fluid path [39]. An efficient arrangement helps to rapidly cool the material, suppress excessive expansion of plant protein fibers, and prevent structural loosening and deformation. It also enables precise control of the cooling rate. This, in turn, promotes the formation of a stable, meat-like fiber network through the interaction of moisture and protein, thereby enhancing the product’s elasticity and chewiness [16,43]. The current mainstream flow channel arrangements include straight-line and serpentine designs. The former uses parallel channels, offering low flow resistance and uniform temperature distribution, making it suitable for rapid cooling of thin-walled or low-viscosity materials. The latter, with its curved paths, extends the heat transfer time, making it more suitable for uniform cooling of high-viscosity or thick-section products.

#### 3.4.4. Summary of Die Design Scheme

**Figure 9:**
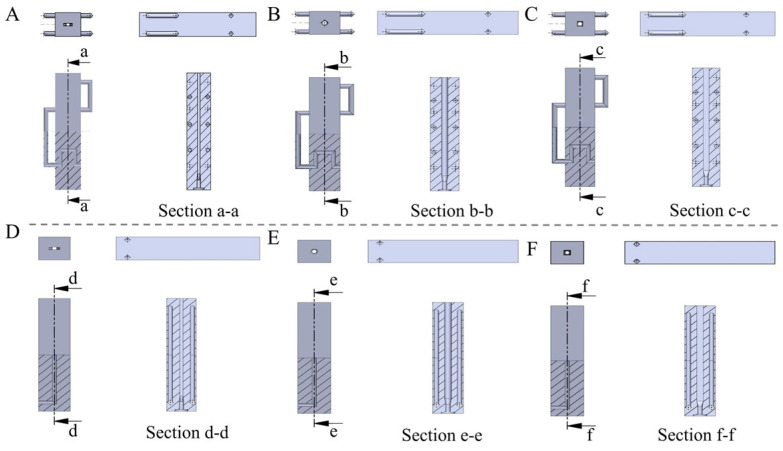
Schematic diagram of serpentine-shaped dual-channel and parallel linear cooling dies. (**A**) rectangular cross-section, (**B**) circular cross-section, (**C**) square cross-section, (**D**) rectangular cross-section, (**E**) circular cross-section, and (**F**) square cross-section.

Based on the high-moisture plant protein extrusion process requirements and the specific design flow and specifications for the cooling die, six cooling die design solutions were developed. These solutions were formulated in conjunction with the actual Twin Lab-F 20/40 twin-screw extruder. These are illustrated in Figure 9. The die body dimensions were designed as 318 mm × 70 mm × 65 mm, which match the inner diameter of the twin-screw extruder barrel. The die cross-section of 70 mm × 65 mm ensures sufficient space for the arrangement of cooling water pipes within the die while minimizing the die weight. The total die length is set to 318 mm, meeting the gradient cooling requirements from the high-temperature zone to the cooling zone for high-moisture protein melt.

For ease of disassembly, cleaning, and daily maintenance, the die is divided into three sections, with each section having specific dimensions of 106 mm × 70 mm × 65 mm, and the sections are connected by screws. The cooling channel cross-section design is based on the extrusion flow rate, with the equivalent cross-sectional area determined to be 168 mm^2^ using the extruder material flow equation. Based on this, three geometric designs were developed: rectangular (24 mm × 7 mm), circular (φ, diameter = 14.64 mm), and square (12.96 mm × 12.96 mm).

For the cooling water pipeline layout, two designs were adopted: parallel straight-line and serpentine dual-channel configurations. The six cooling die design solutions are shown in the Figure 9.

### 3.5. Numerical Simulation Analysis of Different Cooling Die Design Schemes

The cooling die is located at the end of HME. Its core functions include shaping the plant protein melt extruded under high temperature and pressure. Additionally, it induces the ordered arrangement of protein chains during the cooling process. Through these functions, plant-based meat is endowed with a textured structure similar to that of animal muscle [3,44].

For different outlet cross-sections (rectangular, square, and circular) and flow channel layout forms (straight and serpentine), a three-dimensional cooling die model was established. Combined with the measured heat transfer coefficients and temperature-dependent viscosity curves, multi-physics field visualization simulations and predictions were conducted. These focused on the three-stage evolutionary behavior (“solidification-shaping-fibrosis”) of PPI during the extrusion molding process. Comprehensive numerical simulation results show that the geometry of the cooling die and the arrangement of cooling water channels produce a coupling effect in the temperature-rheology-dynamics fields. This coupling effect systematically determines the entire process of plant protein transformation after HME, with detailed quantitative comparisons summarized in Table 3.

**Figure 10:**
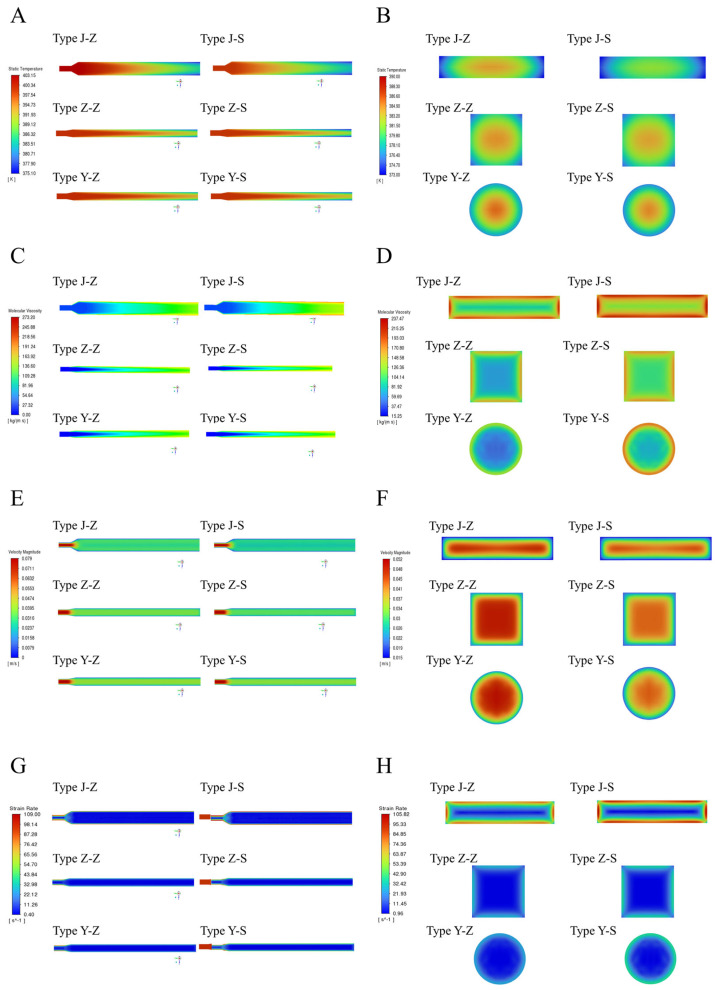
Simulation analysis of PPI flow characteristics under different cooling dies design schemes. (**A**) temperature distribution of flow channel cross-sections, (**B**) temperature distribution of outlet cross-sections, (**C**) viscosity distribution of flow channel cross-sections, (**D**) viscosity distribution of outlet cross-sections, (**E**) fluid velocity distribution of flow channel cross-sections, (**F**) fluid velocity distribution of outlet cross-sections, (**G**) fluid shear rate distribution of flow channel cross-sections, and (**H**) fluid shear rate distribution of outlet cross-sections.

First, after the PPI melt enters the cooling zone, the wall surface cools rapidly. This results in a typical “V”-shaped distribution of the flow channel cross-section in all cooling die designs, with higher temperatures in the center and lower temperatures at the edges (Figure 10A). This phenomenon confirms the rule that the cooling process is significantly more efficient near the wall surface compared to the central region [16]. For the die design scheme with a rectangular outlet cross-section, the serpentine water channel provides a prolonged flow path, extending the heat exchange time and causing the “V”-shape to appear at a more forward position. Quantitatively, at the 200 mm section of the rectangular outlet die, the center–wall temperature difference reached 12.4 °C, compared with 7.8 °C for the circular die. Its average heat transfer coefficient was 128 W/m^2^·K, approximately 35% higher than that of the circular die (95 W/m^2^·K) (Figure 10A). Furthermore, due to its larger perimeter and heat exchange area, the rectangular outlet cross-section further enhances the overall cooling effect (Figure 10B).

Due to the abrupt change in viscosity induced by the temperature gradient, in the rectangular die, the central fluid first enters the viscoelastic transition zone. This forms a low-viscosity “U”-shaped band that contracts axially. In contrast, in the square and circular dies, the low-viscosity regions extend over a longer distance (Figure 10C,D). This fully illustrates the key role of the mechanism where temperature drops and viscosity increases in regulating the overall flow field morphology [45]. This U-shaped viscosity distribution was further quantified by comparing viscosity at the channel center and near-wall regions. As shown in Table 3, the viscosity in the rectangular die increased from 6.2 ± 0.1 Pa·s (center) to 45.8 ± 0.8 Pa·s (near wall), yielding a wall-to-center ratio of 7.4. Similarly, the square and circular dies showed ratios of 4.9 and 3.7, respectively. These data clearly confirm the strong viscosity gradient across the channel, supporting the proposed U-shaped distribution.

The initial increase in viscosity suppresses the axial flow velocity. This results in the largest center-to-wall velocity difference at the outlet of the rectangular die (0.024 m s^−1^) and the smallest in the circular die (0.019 m s^−1^) (Figure 10E,F). This difference is consistent with the corresponding viscosity stratification, where the wall-to-center viscosity ratio of the rectangular die reached 7.4, compared to 4.9 for the square die and 3.7 for the circular die. Such coupled effects determine the sharpness of the Hagen–Poiseuille profile: the greater the velocity and viscosity gradients, the stronger the induced shear forces. Consequently, a larger velocity difference directly correlates with a higher final fibrosis coefficient, aligning with the experimental findings of Guyony et al. [42] who reported a linear relationship between fiber angle and die size. Therefore, protein products produced using the rectangular cross-section die tend to exhibit a texture more similar to that of meat compared with those manufactured using other geometries [46].

In the shear rate field, all designs exhibit a distribution that is low at the center and high at the wall (Figure 10G). Quantitatively, the rectangular die produced the highest wall shear rate, reaching 3.42 s^−1^, whereas the circular die exhibited the lowest at 2.15 s^−1^ (Figure 10H). This trend reflects the combined effects of enhanced cooling and reduced flow velocity in the rectangular geometry. Additionally, the right-angle geometry of the rectangular and square dies induced flow separation in the corners, reducing the local shear rate to approximately 0.96 s^−1^. This reduction further amplified the wall–corner shear gradient, strengthening the potential for localized molecular alignment. Such behavior is consistent with the peak shear concentrations observed by Kern et al. [47] on the upper and lower walls of the flat die. Notably, under the same cross-section, the serpentine water channel generates higher wall shear than the straight one. This is because its enhanced heat exchange reduces the edge flow velocity and increases local viscosity, thereby amplifying the shear gradient. This phenomenon is consistent with the numerical prediction in the field of metal extrusion that “conformal cooling” improves the thermal efficiency of the die [48,49].

In summary, the scheme featuring a rectangular outlet and a serpentine water channel constructs a specific flow field structure. This structure is most conducive to PPI chain orientation and the coordinated solidification of layers and fibers. It achieves this effect by maximizing the wall heat transfer coefficient, viscosity jump, and dual gradients of velocity and shear. This can significantly improve the texturization degree and meat-like simulation performance of high-moisture plant proteins.

## 4. Conclusions

This study investigates the impact of various cooling die designs on the flow characteristics of PPI, with the aim of optimizing the texture of PPI extrudates. Numerical simulations reveal that the cooling die configuration significantly influences the fiber formation process of PPI extrudates. The rectangular outlet with a serpentine cooling channel was the most effective, producing a center-to-wall temperature difference of 12.4 °C, a 35% higher heat transfer coefficient than the circular die, a wall-to-center viscosity ratio of 7.4 compared with 4.9 and 3.7 for the square and circular dies, and a maximum wall shear rate of 3.42 s^−1^ compared with 2.15 s^−1^ for the circular die. These steep thermal and rheological gradients promoted molecular alignment and fiber formation, confirming the rectangular–serpentine cooling die configuration as the optimal design. Future studies should validate these simulation-based predictions through real extrusion experiments and extend this approach to other cooling die designs and plant protein sources to enhance the robustness and applicability of the findings.

## Figures and Tables

**Table 1:** Mesh independence test results for outlet average velocity and centerline temperature.

Mesh Density	Number of Elements (×10^6^)	Outlet Average Velocity (m/s)	Centerline Temperature (°C)	Difference vs. Finer Mesh (%)
Coarse	0.420	0.220	141.200	5.800
Medium	0.780	0.220	143.100	2.100
Fine	1.150	0.230	143.900	0.000

The percentage difference is calculated using the centerline temperature compared to the next finer mesh.

**Table 2:** Results of fitting the power-law model of PPI with *K* and *n* values at different temperatures.

Temperature (°C)	*K* (Pa·s*^n^*)	*n* (Dimensionless)	R^2^
35	236.100 ± 2.450 ^a^	0.200 ± 0.010 ^e^	0.999
55	102.350 ± 1.450 ^b^	0.280 ± 0.020 ^d^	0.997
75	65.200 ± 1.480 ^c^	0.400 ± 0.050 ^c^	0.991
95	47.680 ± 0.390 ^d^	0.430 ± 0.010 ^ab^	0.998
115	17.970 ± 0.220 ^e^	0.440 ± 0.010 ^a^	0.996
135	9.840 ± 0.100 ^f^	0.430 ± 0.020 ^ab^	0.998

Note: Different lowercase letters indicate significant differences (*p* < 0.05).

**Table 3:** Quantitative comparison of cooling die designs in terms of temperature, viscosity, velocity, and shear rate fields.

Parameter	Rectangular Die	Square Die	Circular Die
Center–wall ΔT at 200 mm (°C)	12.400	10.200	7.800
Average heat transfer coefficient (W/m^2^·K)	128.000	112.000	95.000
Viscosity at center (Pa·s)	6.200 ± 0.100 ^a^	7.400 ± 0.200 ^b^	8.100 ± 0.200 ^c^
Viscosity at wall (Pa·s)	45.800 ± 0.800 ^a^	36.200 ± 0.600 ^b^	30.000 ± 0.500 ^c^
Wall/center viscosity ratio	7.400	4.900	3.700
Center–wall velocity difference (m/s)	0.024	0.021	0.019
Max. wall shear rate (s^−1^)	3.420	2.870	2.150
Corner shear rate (s^−1^)	0.960	0.960	1.120

Note: ΔT refers to the temperature difference between the center and wall at 200 mm. Different lowercase letters indicate significant differences (*p* < 0.05).

## Data Availability

The data presented in this study are available on request from the corresponding author.
